# Blastic plasmacytoid dendritic-cell neoplasia: a challenging case report

**DOI:** 10.1007/s00432-021-03777-2

**Published:** 2021-09-16

**Authors:** Ruth-Miriam Koerber, Stefanie A. E. Held, Maria Vonnahme, Georg Feldmann, Joerg Wenzel, Ines Gütgemann, Peter Brossart, Annkristin Heine

**Affiliations:** 1 Medical Clinic III for Oncology, Hematology, Immune-Oncology and Rheumatology, University Hospital Bonn, Venusberg Campus 1, 53127 Bonn, Germany; 2 Department of Dermatology and Allergy, University Hospital Bonn, Venusberg Campus 1, 53127 Bonn, Germany; 3 Department of Pathology, University Hospital Bonn, Venusberg Campus 1, 53127 Bonn, Germany

**Keywords:** Blastic plasmacytoid dendritic cell-neoplasms (BPDCN), Myeloid neoplasia, MDS, AML, Tagraxofusp

## Abstract

Blastic plasmacytoid dendritic-cell neoplasm (BPDCN) is an extremely rare disease that originates from dendritic cells and is associated with a poor overall survival (OS). Diagnostic and therapeutic standards are less well-established in comparison to other leukemic conditions and standards of care are lacking. Morphologic and molecular similarities to acute myeloid leukemia (AML), myelodysplastic syndrome (MDS) and chronic myelomonocytic leukemia (CMML) are hard to distinguish. We here report a BPDCN patient with a long, challenging diagnostic period. While bone marrow biopsies initially failed to prove the correct diagnosis, a cutaneous biopsy finally identified a CD45^+^/CD56^+^/CD4^+^/CD123^+^/CD33^+^/MPO^−^ population suggestive of BPDCN which was confirmed by flow cytometry. Molecular analysis revealed an *ASXL-1, TET2* and *SRSF2*-mutation, cytogenetic analysis showed a normal karyotype. Treatment with the recently approved CD123-cytotoxin Tagraxofusp showed initially a very good response. This case reflects diagnostic and therapeutic difficulties in BPDCN as very rare, easily misdiagnosed neoplasia and the need for precise diagnostic care.

## Introduction

Blastic plasmacytoid dendritic cell neoplasm is a rare hematologic malignancy known to be derived from the precursors of plasmacytoid dendritic cells (pDC) (Bueno et al. 2004). Normal pDCs account for less than 0.4% of peripheral blood mononuclear cells and few of these cells reside in primary and secondary lymphoid organs (Swerdlow et al. 2017). Functional and non-malignant pDCs are typically lineage (Lin)^−^, HLA-DR^+^, CD56^−^, CD123^+^, CD11c^−^, whereas, BPDCN characteristically express CD56 and CD4 (Jegalian et al. 2009). BPDCN have morphologic and molecular similarities to monocytic myeloid leukemias and myelodysplastic syndromes (Riaz et al. 2014). Dysplastic changes can be found in BPDCN, predominantly in the megakaryopoiesis (Laribi et al. 2016). The etiology of BPDCN is unknown, though a preceding, concurrent or subsequent MDS, CMML or AML is possible. Clinical manifestations are often cutaneous lesions, leukemic dissemination and bone marrow (BM) involvement. Patients are generally older than 60 years and have a poor prognosis ∼1 year (Pagano et al. 2013).

## Case description

In August 2020, a 68-year old Caucasian male presented in our clinic with leukocytosis, anemia, fever, dyspnea, dizziness and exhaustion. On examination, he was severely pale with no signs of peripheral lymph node enlargement, evidence of bleeding or jaundice. The chest was clinically clear, liver and spleen were not palpable.

Automated blood count showed high leukocyte counts (34.5 G/L), severe normochromic, normocytic anemia (4.2 g/dl) and normal platelets (209 G/L). Iron stores and Vitamin B12 were in normal ranges, whereas folic acid was slightly reduced (3.94 ng/ml). Blood smears showed 3% blasts, lymphopenia (16%) and neutrophilia (71%). Monocyte counts were normal (1%). Blood counts were spontaneously fluctuating on a monthly basis, altering to pancytopenia, recovering to normal levels and again leukocytosis.

**Fig. 1:**
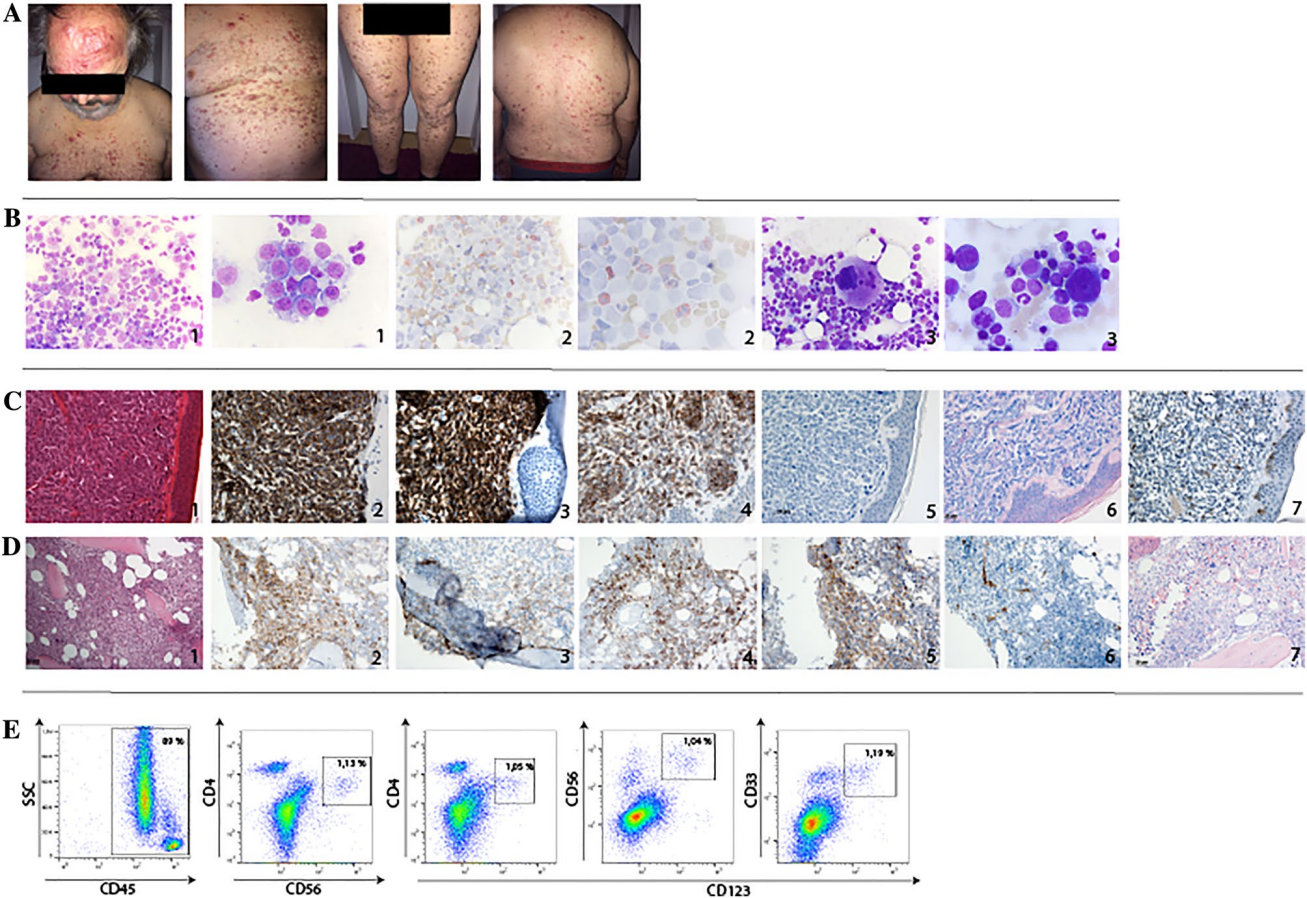
**a** Images of cutaneous lesions of the patient with BPDCN. Multiple cutaneous nodules and papules spread over the whole body. **b** Bone marrow smears taken from the patient with BPDCN. (1) Pappenheim staining of a bone marrow smear taken at diagnosis of BPDCN showing infiltration of large cells, with a wide basophilic cytoplasm and blastic nucleus partially with nucleoli. Approximately 30% infiltration (magnification 50× and 100×). (2) Peroxidase staining of a bone marrow smear exhibiting only positivity in granulopoiesis, BPDCN blasts are negative. (3) Dysplastic features of megakaryopoiesis and erythropoiesis (magnification 50× and 100×). **c** Immunephenotype of BPDCN. IHC staining of cutaneous lesions: (1) HE-staining (20×), (2) CD3, (3) CD56, (4) CD123, (5) MPO, (6) Giemsa 40×, (9) CD117. (D) IHC staining of bone marrow showed diffuse infiltration of the BPDCN: (1) HE-staining 20×, (2) CD4, (3) CD56, (4) CD123, (5) CD33, (6) CD34, (7) Giemsa 40×. (E) Flow cytometric analysis of peripheral blood showed, in contrast to BM, only a small distinct CD45^+^, CD123^+^, CD4^+^, CD56^+^, CD33^+^ population. Figure was created using Adobe Illustrator CC 2019

**Fig. 2:**
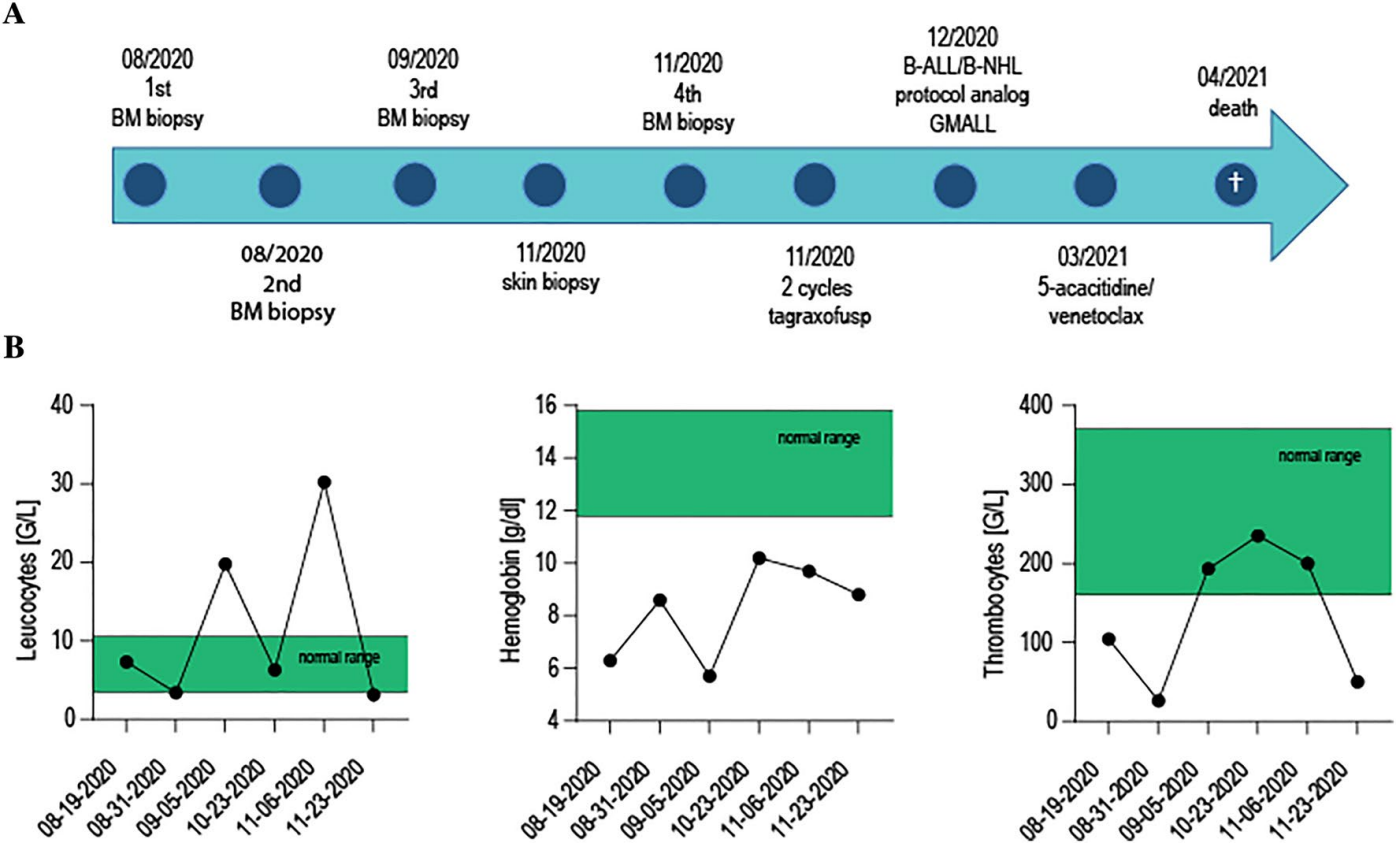
Clinical course of diagnostic interventions and treatment schedule. The timeline shows the sequence of the diagnostic period and the different treatment regimes. Fluctuating blood counts during the diagnostic period are shown for absolute leukocyte- and thrombocyte counts and hemoglobin levels

An initial BM biopsy was suggestive for myelomonocytic, acute leukemia. We repeated the aspiration a couple of days later and found a 30% infiltration of large cells with a wide basophilic cytoplasm and a blastic nucleus partially with nucleoli. Cells did not have microvacuoles or pseudopodia. POX- and esterase-staining were negative. Cells were both found disseminated and clustering (Fig. 1B). The overall appearance was not typical for an AML, ALL or multiple myeloma. Standard immunophenotyping showed no typical blasts or suspicious lymphoid populations. Paraffin-sections could also not verify the external AML diagnosis. Classical mutations for AML or myeloproliferative neoplasms were negative. Immunohistochemistry ruled out BM carcinosis, but showed a distinct CD56^+^ population. A reference pathologist could also not further classify this population. The karyogram was normal. NGS-analysis showed *ASXL-1, TET2* and *SRSF2*-mutations. Since no definite diagnosis could be made, we repeated BM biopsy one month later. Here, the previously described CD56^+^ population was not apparent anymore. CT-scans gave no evidence of lymphadenopathy, hepatosplenomegaly or aspects of a solid tumor. Meanwhile, our patient developed multiple cutaneous reddish plaques (Fig. 1A). Histopathologic examination showed a diffuse, monomorphic infiltrate of medium-sized blasts with irregular nuclei, plenty mitoses and a small cytoplasm. The entire dermis was infiltrated, sparing the epidermis. CD45 positivity proved leukocytic origin, suitable for cutaneous chloroma. Further immunohistologic stainings showed positivity for CD4, CD56, CD33, CD123 and partially CD38. Progenitor markers like CD117 showed only weak to no expression, CD34 was negative. MPO, CD20 and CD10 were also negative. Ki-67 staining revealed a high proliferative index (70%). Therefore, we repeated the BM biopsy and found similar cells like in the first biopsy and in the skin. Furthermore, signs of dysplasia, predominantly in the megakaryopoiesis and erythropoiesis, were present. Immunophenotyping and immunohistology also revealed the same phenotype as the cutaneous lesion (Fig. 1C–E). Semi-nested PCR ruled out monoclonal rearrangement of the B-/T-cell receptor. Therefore, the diagnosis of BPDCN with extramedullary manifestation (skin) could be made. Especially, distinction of acute myelomonocytic leukemia was difficult, since these cases can also express CD33, CD123 and aberrantly CD56, but are typically positive for MPO. Though NGS-analysis (Skin and BM) detected *ASXL-1, TET2* and *SRSF2-*mutations, secondary AML due to MDS seemed unlikely, since dysplastic features were apparent in less than 50% of non-blasts (Fig. 2).

Intensive examinations for differential diagnosis were conducted, but all were negative (viral/bacterial/fungal infections, hemoglobin-anomalies, paroxysmal nocturnal hemoglobinuria, pure red cell aplasia, hemolysis and alcohol abuse).

We started treatment with the recently FDA-approved CD123 cytotoxin Tagraxofusp (Elzonris®) (Pemmaraju 2020) in November 2020. Due to the poor prognosis and high-risk cytogenetic mutations, a hematopoietic allogenic stem cell transplantation was planned in first remission (Ohe et al. 2018). Unfortunately, Tagraxofusp had to be discontinued after two cycles due to inacceptable hepatic toxicity and a relevant capillary leak syndrome, though clinically primary treatment response seemed to be very good. Therapy was switched to an aggressive B-ALL/B-NHL protocol analog GMALL 2002 (Garnache-Ottou 2019) in the end of December 2020. Again, progressive disease in March 2021 forced us to switch the regimen. We next decided to treat with 5-acacitidine/venetoclax, since in vitro studies show dependency on Bcl-2 of BPDCN cells (Montero et al. 2017) and clinical trials are ongoing (NCT03485547). Unfortunately, although this change of treatment approach, our patient developed again progressive disease and died in a fulminant neutropenic, septic shock. The clinical time course is shown in Fig. 2.

## Methods

Patient samples were withdrawn for routine diagnostic tests. Cytology stainings, immunophenotyping on a Beckmann Coulter Navios EX and NGS analysis on Illumina MiniSeq platform were performed in our department. Photographies of the patients were taken after informed consent. Histomorphology and immunohistologic stainings were performed in the local Department of Pathology.

## Discussion

BPDCN is associated with comprised overall survival, and clinical standards are missing (Swerdlow et al. 2017). In the past, nomenclature of BPDCN frequently changed. Initially, BPDCN was listed as AML, in 2016 the WHO reclassified its own category (ancient names include: acute-agranular-NK-cell-leukemia, blastic-NK-cell-lymphoma, CD4^+^CD56^+^-hematodermic neoplasm) (Vardiman et al. 2009). Struggles in nomenclature display clinical diagnostic difficulties.

Access of targeted therapies requires correct clinical diagnostics, to supply patients with sufficient therapies. To distinguish other hematologic malignancies with cutaneous involvement from BPDCN, T-/NK-cell neoplasms and extramedullary myeloid sarcoma (EMS) must be ruled out. Cutaneous T-cell lymphoma rather infiltrates the epidermis and lacks CD56 expression. Extranodal NK-cell malignancies share common features of BPDCN, though these are typically EBER (Epstein-Barr-encoding region) positive. Furthermore, rearrangement of the B-/T-cell receptor genes can help to distinguish BPDCN from lymphoid malignancies (Ishida and Kwong 2010). To help making a correct BPDCN diagnosis we compiled Table 1 (Alayed et al. 2013; Swerdlow et al. 2017; Ishida and Kwong 2010).

**Table 1:** Current markers are listed to delimit BPDCN from other myeloid malignancies

	Typical for BPDCN	Common in BPDCN		Typical for BPDCN	Common in BPDCN
T-cell marker			B-cell marker		
CD4	+	+	CD19	−	−
CD3	−	−	CD20	−	−
CD5	−	−	CD79a	−	−
CD7	−	+	CD38	−	−/ +
Myeloid marker			pDC-markers		
CD33	−	+	CD123	+	+
CD13	−		BDCA2 (CD303)	+	+
CD14	−		BDCA4 (CD304)	+	+
CD45	+	+	CD2AP	+	+
MPO	−	−	Spi-B	+	+
NK-cell marker					
CD56	+	+	Other		
Progenitor-/activation-marker	−	−	TCL-1	+	+
CD34	−	−	CD31	+	+
CD117	−/ +	−/ +	EBER	−	−
Tdt	−	−/ +	CD43	+	+
HLA-DR	+	+	CD11c	−	−
			Lysozyme	−	dim

Especially CD56 helps to distinguish reactive from malignant pDCs, though rare cases of CD56 negative BPDCNs have been described

Common somatic mutations in AML and MDS like *ASXL-1, TET-2, TP53, IDH1/2, NPM1, DNMT3A* and *NRAS* can be observed in BPDCN as well. BPDCN usually have multiple karyotypic abnormalities, though these are not specific for diagnostics (Menezes et al. 2014). Further shared features of MDS/CMML like dysplasia can be observed in BPDCN. Whether this is expression of an underlying MDS/CMML, or a primary BPDCN feature can yet not fully be defined. Probably, only the medical history of patients (preexisting cytopenia, monocytosis etc.) is helpful.

Finally, our case shows typical difficulties of BPDCN diagnosis and treatment. In contrast to published data, the diagnostic time of almost 4 months was not dominated by an aggressive disease, though this changed with flaring of the cutaneous lesions. The reason for periodic fluctuation of blood counts, observed from August until November 2020, is not well-understood. Retrospective interpretation of the first biopsy was also suggestive of BPDCN infiltration, therefore, an underlying MDS/CMML was unlikely.

## Conclusion

Taken together, this case represents the difficulties in rare malignant hematologic disease and the need for expertise of hematologists, pathologists and laboratory medicine personnel. Patients presenting with skin symptoms should be evaluated for primary hematologic disease, especially if concomitant blood count abnormalities or lymphadenopathy are present. BPDCN must be distinguished from myelomonocytic acute leukemia and T-/NK-cell neoplasms. Characteristic phenotypic surface markers like CD123 or CD303 are not established in most of the diagnostic routine laboratories and specific cytogenetic abnormalities do not exist. Since a new targeted therapy for BPDCN is now available, physicians should be aware of this rare disease.

## Data Availability

The datasets generated during and/or analyzed during the current study are available from the corresponding author on reasonable request.
